# Supplementary material for: Content-rich biological network constructed by mining PubMed abstracts
Source: BMC Bioinformatics. 2004 Oct 8;5:147. doi: 10.1186/1471-2105-5-147 (PMC528731; doi:10.1186/1471-2105-5-147)
Supplement: Additional File 2 — The original results of the above study (non-essential files are deleted to keep the file size under the limit set by BMC bioinformatics). [file 1471-2105-5-147-S2.bz2 › chilibotAdditionalFile2/dip20/669ID11110785E11071/html/CCR3_CXCL9.html]

 


 **CCR3** and **CXCL9** 
  
Found 20 abstracts in PubMed, retrieved 20.  
 

 What does Google say? 
 PDF only 
| .edu only 

---

**Interactive relationship** (e.g. stimulation, inhibition, etc)

**Neutral relationship**- **CCR3**  functional responses are regulated by both CXCR3 and its ligands  **CXCL9** , CXCL10 and CXCL11.  Ref: 12884299 Eur J Immunol, 2003
- The ligands of CXC chemokine receptor 3  [ **CCR3** ] , I TAC, Mig  [ **CXCL9** ] , and IP10, are natural antagonists for  **CCR3** .  Ref: 11110785 J Biol Chem, 2001
- Immunohistochemical staining was performed using antibodies for CXCR3 a specific receptor for IP10 and MIG  [ **CXCL9** ] , which is characteristic of Th1 helper phenotype and  **CCR3** , CCR4 and ST2, which are features of Th2 cells.  Ref: 11920617 Int J Cancer, 2002
- IP10, MIG  [ **CXCL9** ]  and TARC positive HD cases contained higher numbers of Th2 lymphocytes ST2  **CCR3**  or CCR4 positive lymphocytes, compared to IP10, MIG  [ **CXCL9** ]  and TARC negative HD cases p <.  Ref: 11920617 Int J Cancer, 2002
- I TAC, Mig  [ **CXCL9** ] , and IP10 compete for the binding of eotaxin to  **CCR3**  bearing cells and inhibit migration and Ca 2 changes induced in such cells by stimulation with eotaxin, eotaxin 2, MCP 2 monocyte chemottractant protein 2, MCP 3, MCP 4, and RANTES regulated on activation normal T cell expressed and secreted.  Ref: 11110785 J Biol Chem, 2001
- IP 10 and Mig  [ **CXCL9** ]  share a common functional receptor on activated T lymphocytes, named CXC chemokine receptor 3  [ **CCR3** ]  CXCR3.  Ref: 11134180 J Clin Invest, 2001
- Type 1 chemokines CXC chemokine ligand CXCL 9  [ **CXCL9** ]  and CXCL10 monokine induced by IFN gamma and IFN gamma inducible protein of 10 kDa CXCR3 ligands, and type 2 chemokines CC chemokine ligand CCL 11 eotaxin  **CCR3**  ligand, CCL17 thymus and activation regulated chemokine CCR4 ligand and CCL22 monocyte derived chemokine CCR4 ligand were readily detectable in the plasma of most participants.  Ref: 12407016 Int Immunol, 2002
- CXC chemokine receptor 3  [ **CCR3** ]  CXCR3, predominately expressed on memory activated T lymphocytes, is a receptor for both IFN gamma inducible protein 10 gamma IP 10 and monokine induced by IFN gamma Mig  [ **CXCL9** ] .  Ref: 10903763 J Immunol, 2000
- CXC chemokine receptor 3  [ **CCR3** ]  CXCR3, which is known to be expressed predominately on memory and activated T lymphocytes, is a receptor for both interferon gamma IFN gamma inducible protein 10 gamma IP 10 and monokine induced by IFN gamma Mig  [ **CXCL9** ] .  Ref: 10942362 Blood, 2000
- In contrast, monokine induced by IFN gamma HuMig  [ **CXCL9** ] , which like HuIP 10 is an agonist of C X C chemokine receptor 3  [ **CCR3** ] , does not inhibit KSHV GPCR signaling.  Ref: 9670053 J Exp Med, 1998
